# Unexpected benzene oxidation in collisions with superoxide anions

**DOI:** 10.1038/s41598-021-02408-7

**Published:** 2021-11-30

**Authors:** Carlos Guerra, Sarvesh Kumar, Fernando Aguilar-Galindo, Sergio Díaz-Tendero, Ana I. Lozano, Mónica Mendes, Paulo Limão-Vieira, Gustavo García

**Affiliations:** 1 Instituto de Física Fundamental, Consejo Superior de Investigaciones Científicas, Serrano 113-bis, 28006 Madrid, Spain; 2 Atomic and Molecular Collisions Laboratory, CEFITEC, Department of Physics, Universidade NOVA de Lisboa, 2829-516 Caparica, Portugal; 3 Donostia International Physics Center (DIPC), Paseo Manuel de Lardizabal 4, 20018 Donostia-San Sebastián, Spain; 4 Departamento de Química, Universidad Autónoma de Madrid, Módulo 13, 28049 Madrid, Spain; 5 Condensed Matter Physics Center (IFIMAC), Universidad Autónoma de Madrid, 28049 Madrid, Spain; 6 Institute for Advanced Research in Chemical Science (IAdChem), Universidad Autónoma de Madrid, 28049 Madrid, Spain; 7 Centre for Medical Radiation Physics, University of Wollongong, Wollongong, NSW Australia

**Keywords:** Atomic and molecular physics, Plasma physics

## Abstract

Superoxide anions colliding with benzene molecules at impact energies from 200 to 900 eV are reported for the first time to form massive complexes. With the aid of quantum chemistry calculations, we propose a mechanism in which a sudden double ionization of benzene and the subsequent electrostatic attraction between the dication and the anion form a stable covalently bonded C_6_H_6_O_2_^+^ molecule, that evolves towards the formation of benzene-diol conformers. These findings lend support to a model presenting a new high energy anion-driven chemistry as an alternative way to form complex molecules.

## Introduction

Benzene is one of the simplest and more stable aromatic ring molecules. It has been considered as a prototype for the study of chemical reactions involving biomolecules (pyridine^1^, carbamates^2^), a building block for the synthesis of carbon nano-cages^3^ and a precursor of hydrocarbon structures (phenol, toluene, aniline, anthracene) with important technological applications^4^. Oxygen superoxide anion (O_2_^−^) is one of the reactive oxygen species (ROS) which is responsible for several biochemical processes leading to oxidative damage in living organisms^5^ and materials^6^. In particular, 8-oxoguanine is frequently formed by the interaction of ROS with the guanine base in DNA under conditions of oxidative stress yielding an efficient way of damaging DNA^7^.

Oxidative processes in benzene have been extensively studied from different points of view of scientific and technical relevance, including e.g. atmospheric reactions^8,9^, surface catalyzed reactions^10^ or direct mechanisms induced by metallic oxide cations^11^. Most of these procedures fall into the domain of room temperature chemical reactions, i.e. no significant kinetic energy of the reactants is required to trigger such processes. Yet, if the kinetic energy involved in these collisions increases, new channels are open yielding excitation, ionization or even molecular dissociation, compatible with the transferred energy. Ascenzi et al.^12^ showed that oxygen-benzene reactions in atmospheric pressure plasmas (corona and dielectric barrier discharges) may induce energetically disfavored chemical processes leading to formation of phenol cations (C_6_H_5_OH^+^) and neutral phenol (C_6_H_5_OH). Additionally, relevant ion chemistry processes in the interstellar medium (ISM) have been reported from low-energy anion induced reactions with different types of hydrocarbons yielding increasingly complex molecules^13^.

Here we show for the first time that gas-phase energetic interactions of a superoxide anion with a benzene molecule lead to an unexpectedly and quite efficient oxidation process of the neutral molecule. Under the current experimental collision energy range probed (200–900 eV), significant neutral and cationic fragmentation of benzene is reported, whilst a comprehensive description of the underlying collision dynamics mechanisms is investigated. In addition, the experimental conditions provide binary collisions between the incoming projectile (O_2_^−^) and the neutral target molecule (C_6_H_6_), where no positive ions with higher mass-to-charge ratio (*m/z*) than that of the parent ion (*m/z* = 78 u) are expected to be formed. However, as we will discuss below, the mass/charge analysis of the collision products reveals the presence of a prominent feature at *m/z* ~ 110 u for specific impact energies between 200 and 900 eV.

The remainder of this paper is organized as follows. In the next section we briefly present our experimental and theoretical methods, then the corresponding results are presented and discussed and finally some conclusions from this study are drawn in the last section.

## Methods

**Figure 1:**
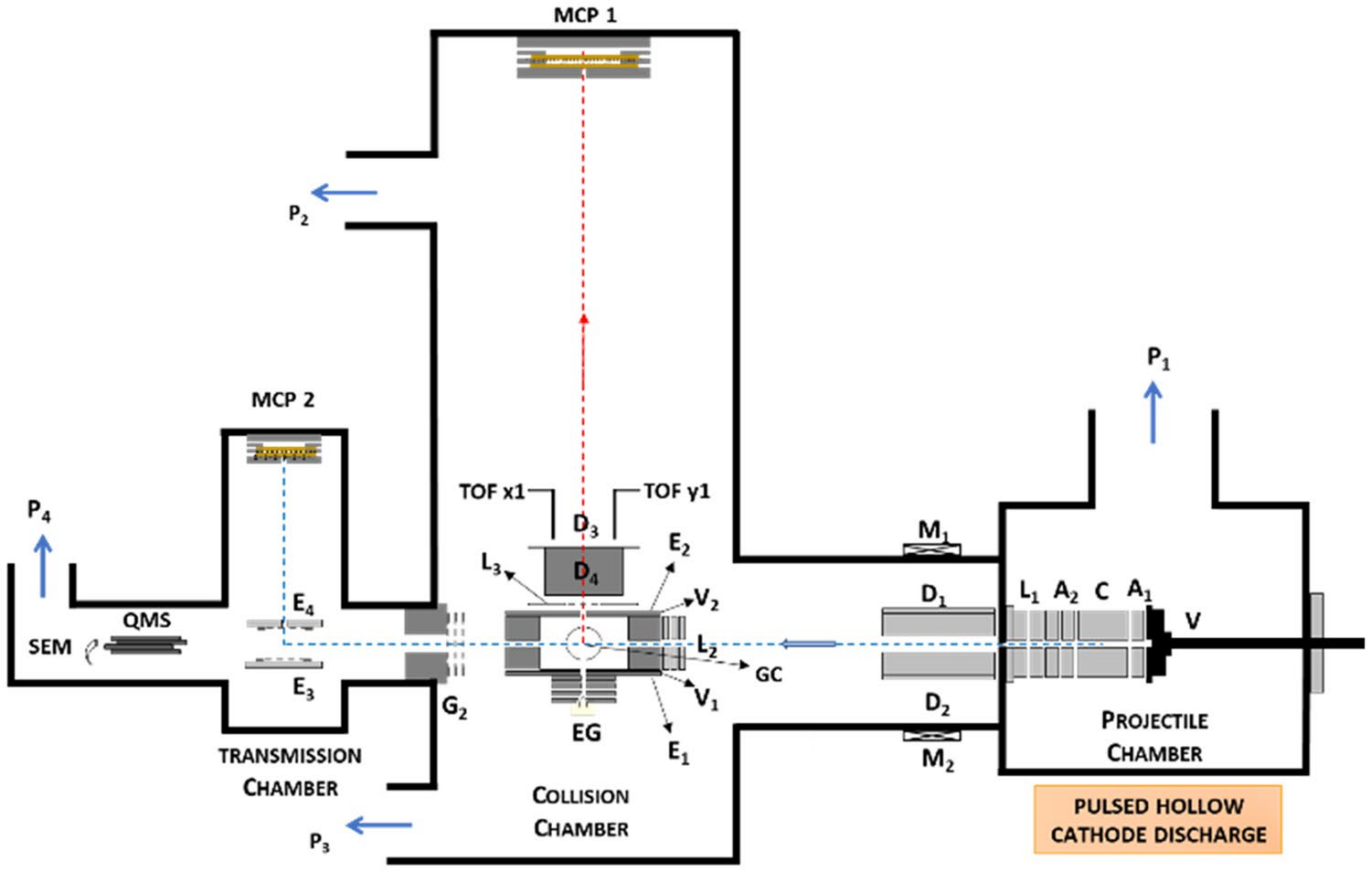
Schematics of the experimental setup. V, pulsed supersonic valve; C, hollow cathode discharge; A1 and A2, anodes (grounded); L1, L2, and L3, Einzel lenses; D1, D2, D3, and D4, deflecting plates; M1 and M2, magnets; E1, E2, E3, and E4, extraction plates; V1 and V2 voltage chamber; G2, retarding field analyzer grids; EG, electron gun; GC, gas cell; MCP 1 and MCP 2, multichannel plate detectors; QMS, quadrupole mass spectrometer; SEM, secondary electron multiplier detector; P1, P2, P3, and P4, turbomolecular pumps.

The experimental arrangement has been reported elsewhere^14^. Briefly, the primary O_2_^−^ beam is generated in the afterglow of a pulsed hollow cathode discharge. A schematic diagram of the experimental set-up is shown in Fig. 1.

**Figure 2:**
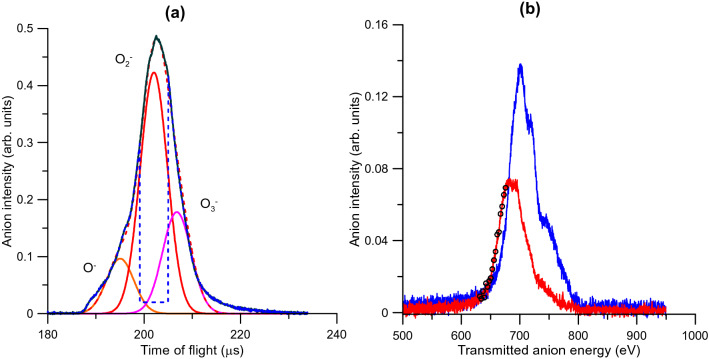
(**a**) Time of flight mass spectra of the primary anion beam: (―) O^−^, (―) O_2_^−^, (―) O_3_^−^, (–-) fraction of the beam removed by the extraction pulse (see text for details). (**b**) Energy spectrum of the transmitted anion intensity for (―) no target molecules in the gas cell, (―) 1.3 mTorr of benzene in the scattering chamber, (o) representative points for which the energy loss of the transmitted O_2_^−^ anions is higher than 24.4 eV (above the double ionization energy limit).

The precursor gas projectile (O_2_) is admitted into vacuum through a commercial Parker pulsed valve (VAC1250) operated at 350-μs pulse-width in an 80-ms duty cycle and at a gas pressure of 4.0 × 10^-5^ mbar. It produces a supersonic expansion neutral beam that triggers the discharge when it passes along the 20 mm long hollow cathode which is negatively biased (-500 V). Positive ions initially formed in the discharge are neutralized during the plasma extinction and negative ions are finally formed by electron attachment processes. Negatively charged particles are extracted from the cathode and focused into the entrance aperture of the collision chamber (electrons are removed from the primary beam with the help of two permanent magnets placed along the beam path). The collision chamber (CC) is a 30 mm length metallic cube that contains the target C_6_H_6_ gas at a well-known pressure (as measured with a MKS Baratron 627DX). A parallel plate system along the axis, inside the collision chamber, allows the extraction of the positive ions formed during the O_2_-C_6_H_6_ interactions by applying a 50–900 V pulse which is synchronized with the control unit of the supersonic valve. Positive ions enter a 1.5 m drift tube and their time-of-flight (TOF) is analyzed with a digital oscilloscope (Tektronix MSO 3034, 2.5 GS/s). Ion signals are formed with a microchannel plate detector (MCP-1) in single pulse mode operation with typical counting rates below 10^2^ s^−1^. Primary particles emerging from the CC, after the interactions, are deflected into a second 0.25 m length TOF spectrometer and detected by another microchannel plate detector (MCP-2). The maximum counting rate of the primary beam was typically 10^3^ s^−1^. The C_6_H_6_ pressure was maintained within 0.1–1.5 mTorr. These conditions ensure a binary collision regime and multiple scattering processes are negligible. Beneath the gas cell, an electron gun provides an energy-controlled electron beam (0–1000 eV) entering the CC normal to the anion beam and opposite to the cation TOF mass analyzer. This electron beam is used to analyze the molecular composition of the background and the gas target as well as to provide a reference fragmentation pattern of the molecular target induced by electron impact^15^. The uncertainty on the point along the 2 cm cathode where anions are produced is spreading the arrival time distribution. This time uncertainty introduces an inherent mass resolution limitation which has been estimated to be Δ*m*/*m* = 0.05. Typical time of flight (TOF) mass spectra, of the primary anion beam (see Ref.^14^ for details), are shown in Fig. 2 for 700 eV. This figure reveals that the anion beam is mainly composed by two species assigned to O_2_^−^ and O_3_^−^. Once the extraction pulse is applied, the area of the primary beam removed from the beam (see Fig. 2a) corresponds to the colliding charged species which generated the extracted ion fragments. By proper tuning the extraction pulse delay, this area can be selected all along the primary beam profile, thus allowing the choice of the projectile to collide with the target. The time scale is energy calibrated for different anion incident energies and the energy distribution corresponding to two different target conditions (0 and 1.3 mTorr of benzene in the scattering chamber, respectively) is plotted in Fig. 2b.

Calculations of the potential energy surfaces were performed in the frame of the density functional theory, in particular using the hybrid long-range corrected CAM-B3LYP functional^16^ in combination with the cc-pVDZ^17^ basis set. This functional shows a variable amount of HF and exchange interactions at short- and long-range and has been shown to predict exited states with better accuracy than other widely used functionals, in particular performing well for charge transfer excitations thanks to the addition of the long-range correction^16,18,19^. We have checked the quality of the basis set by comparing absorption spectra computed with a bigger basis, aug-cc-pVTZ, obtaining energy differences for the states of interest of less than 0.1 eV. In the exploration of the potential energy surface the critical points, i.e. minima and transition states, have been accurately located. Harmonic frequencies were computed to confirm the nature of the minima and the transition states (TSs). Furthermore, intrinsic reaction coordinate calculations were carried out to verify connectivity between TSs and adjacent minima. Harmonic frequencies were also used to correct relative energies with the Zero Point Energy (ZPE). Those simulations involving electronic excited states were performed within the time dependent DFT (TD-DFT)^20,21^, by using the same functional and basis set. We have also carried out a Natural Transition Orbital (NTO) analysis^22^ in the relevant charge transfer excited state. Details on molecular dynamics simulations are given in the Supplementary Information. All simulations have been carried out with the Gaussian16 program^23^.

## Results and discussion

**Figure 3:**
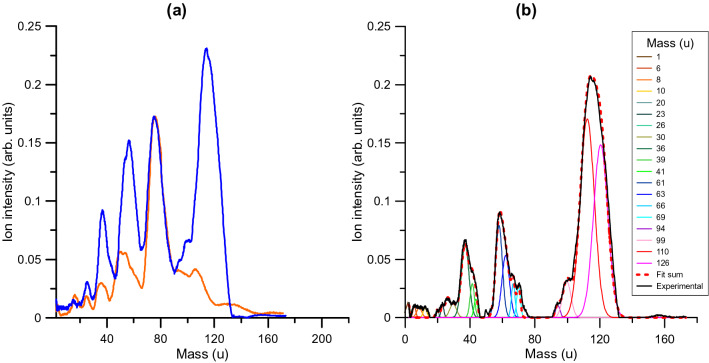
Positive ion spectra. (**a**) cationic fragmentation induced by the electron beam (―) and the negative ion (―) impact. (**b**) mass analysis (Gaussian function fitting) of the positive ion pattern obtained by subtracting the electron impact spectrum from that of the negative ion impact.

A typical TOF spectrum of the cations extracted after the interaction of O_2_^−^ is shown in Fig. 3a for 700 eV incident energy and 1.2 mTorr of benzene in the scattering chamber^14^. The electron impact mass spectrum, generated in such conditions but with a 700 eV electron beam normal to the target sample, is also shown in this figure for comparison. Both spectra are normalized to the parent ion (C_6_H_6_^+^, 78u) intensity. A close inspection of this figure shows that the cation fragmentation induced by the anion beam is qualitatively the same as with the electron beam for *m/z* smaller than 78 u. Notwithstanding the impact energy being the same in both cases, the collision time, projectile velocity and the energy transferred are quite different in both beams, thus lending support to the changes in the yields from both spectra. However, the main differences appear for generated molecular cation species larger than 78 u, where in Fig. 3b we depict the difference between the anion and electron induced fragmentation spectra normalized to the parent ion intensity. This subtraction procedure ensures that features shown in Fig. 3b are only induced by the anion beam without background contributions. In this figure, the most intense feature is found between 110 and 120 u together with other minor contributions at 94 and 99 u.

In order to assign the 110–120 u structure’s composition, we have first proved that for low pressure (< 1.5 mTorr) conditions, only single binary collisions prevail. In this pressure range, no dependence of the measured attenuation cross-section (total electron detachment cross-section) with the gas pressure has been observed so ensuring that multiple scattering processes are negligible. In addition, we have verified that the intensity of the 110–120 u feature remains proportional to that of the parent ion for benzene pressures ranging from 0.1 to 1.5 mTorr. Under these conditions, the only possible assignment for the ion formed after collision of O_2_^−^ and C_6_H_6_ with 110 u, is to consider that the projectile forms with the target a positively charged complex: ${\mathrm{C}}_{6}{\mathrm{H}}_{6}{\mathrm{O}}_{2}^{+}$. Yet, the quite broad nature of the 110–120 u feature shows that it can accommodate an extra contribution (126 u in Fig. 3b) arising from ${\mathrm{C}}_{6}{\mathrm{H}}_{6}{\mathrm{O}}_{3}^{+}$ formation. These weakly bound complex formations are discussed and supported with the aid of quantum chemical calculations in the next paragraphs.

${\mathrm{C}}_{6}{\mathrm{H}}_{6}{\mathrm{O}}_{2}^{+}$ has been previously identified as an adduct generated in air plasmas and has been proposed as a precursor for phenol production^12^. More prominent was the 110 u feature observed by Tubaro et al*.*^24^ in their atmospheric pressure plasma conditions, together with 94 u formation, the latter which was identified as being generated by the loss of an oxygen atom from the former compound according to the following mechanism:

1 $${\mathrm{C}}_{6}{{\mathrm{H}}_{6}^{+\cdot}+{\mathrm{O}}_{2}}\to {\mathrm{C}}_{6}{\mathrm{H}}_{6}{\mathrm{O}}_{2}^{+\cdot}\to \mathrm{O}+{\mathrm{C}}_{6}{\mathrm{H}}_{5}{\mathrm{OH}}^{+\cdot}$$

Although the experimental circumstances of atmospheric plasmas and single collision events in high vacuum are categorically different, it is important to analyze possible reaction mechanisms leading to these species within the current experimental conditions and with the help of theoretical calculations on the underlying mechanisms dictated by the collision dynamics.

For this purpose, we have performed quantum chemical calculations exploring different potential energy surfaces along the reaction coordinate system, i.e. [C_6_H_6_…O_2_]^+^ for the following process:

2 $${\mathrm{O}}_{2}^{-}+{\mathrm{C}}_{6}{\mathrm{H}}_{6}\to {\mathrm{C}}_{6}{\mathrm{H}}_{6}{\mathrm{O}}_{2}^{+}+2{e}^{-}$$

**Figure 4:**
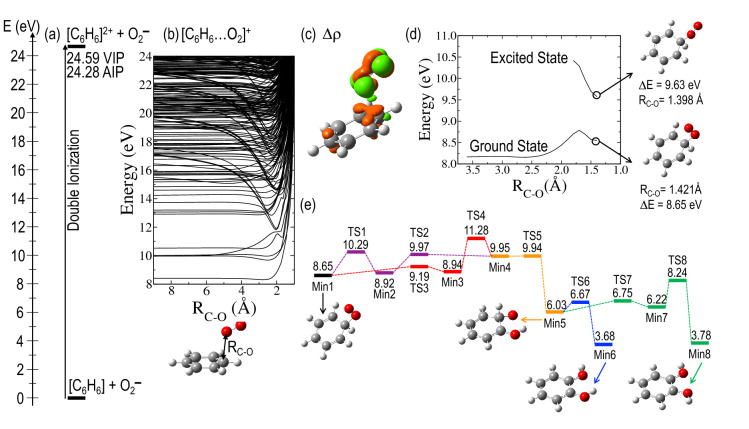
(**a**) 1st + 2nd Ionization energy of benzene, C_6_H_6_, keeping the geometry of the neutral (vertical ionization potential—VIP), and after geometry optimization of the doubly-ionized (adiabatic ionization potential—AIP). (**b**) Potential energy curves as a function of the benzene–oxygen distance for the [C_6_H_6_…O_2_]^+^ system in a frozen scan. (**c**) Distribution of the electronic density difference (∆$\rho$ with an isovalue of 0.007) between the ground and the lowest energy excited state with charge transfer character C_6_H_6_^2+^–O_2_, for R_C-O_ ≈ 2 Å. Red and green lobes correspond to loss and gain of electron density, respectively. (**d**) Potential energy curves as a function of the C-O distance in a relaxed scan both in the ground and in the excited states with charge transfer character. (**e**) Critical points in the potential energy surface of C_6_H_6_O_2_^+^ ground state with doublet spin multiplicity. Min indicates a minimum, TS indicates a transition state. Relative energies are given in eV and have been corrected with the zero-point-energy (ZPE). See rest of the structures in the Supplementary Information. In all panels relative energies are given in eV and refereed to the ground state of C_6_H_6_ and O_2_^−^ at infinite distance.

Differences in charge state and kinetic energy between reactions (1) and (2) are noticeable, but it is interesting to check if, from the theoretical point of view, reaction (2) is feasible in single collisions experiments. Molecular dynamics studies starting from the weakly bound [C_6_H_6_…O_2_]^+^ complex, did not lead to formation of any stable compound with stoichiometry C_6_H_6_O_2_^+^ (see details in the Supplementary Information). Thus, other mechanism should be responsible for the appearance of such cation in the TOF mass spectra. We first investigated the possibility of forming an electrostatically bonded complex by assuming an initial sudden double ionization of benzene by O_2_^−^ impact (see Fig. 4a), according to the reaction:

3 $${\mathrm{O}}_{2}^{-}+{\mathrm{C}}_{6}{\mathrm{H}}_{6}\to {\mathrm{C}}_{6}{\mathrm{H}}_{6}^{2+}+{\mathrm{O}}_{2}^{-}+2{e}^{-}\to {\mathrm{C}}_{6}{\mathrm{H}}_{6}{\mathrm{O}}_{2}^{+}+2{e}^{-}$$

The resulting charged product, C_6_H_6_^2^^+^ and the projectile O_2_^−^ will be attracted by an electrostatic force, thus leading a cationic complex of the form [C_6_H_6_…O_2_]^+^ to be produced. Double ionization of benzene by electron impact has been recently studied by Wolff et al*.*^25^, showing an appearance energy of about 27 eV, which is in reasonably good agreement with the vertical double ionization energy of 24.6 eV, and the yield of formation, with respect to the parent ion, reaches a maximum value of about 5% around 100 eV electron impact energy. More recently Sigaud and Montenegro^26^ found this percentage to be higher than 10% and highlighted that benzene has a much larger double ionization cross section than other studied molecules^26^. In case of a ${\mathrm{O}}_{2}^{-}$ projectile, this yield can be much more efficient. In fact, the energy spectrum of the beam transmitted through 1.3 mTorr of benzene, in Fig. 2b, shows an average energy loss with respect to the initial beam of about 16 ± 3 eV. This demonstrates that the 700 eV anion beam is able to excite, ionize or even double ionize benzene without losing the attached electron.

**Figure 5:**
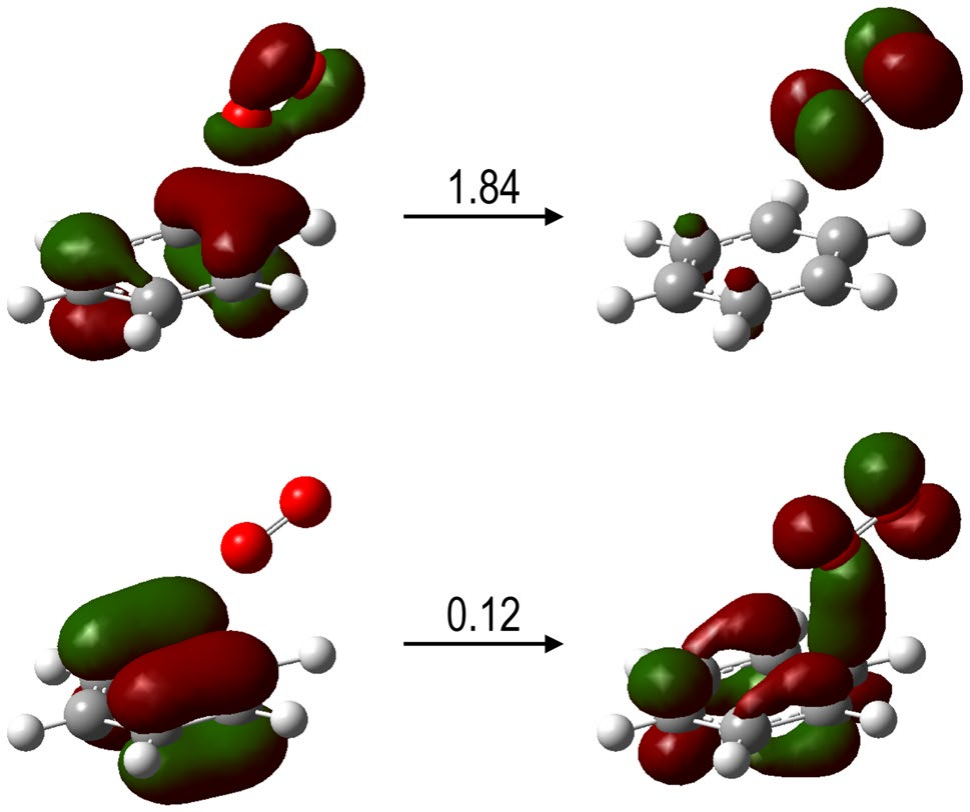
Natural transition orbitals (NTOs) analysis of the lowest energy excited state with charge transfer character C_6_H_6_^2+^–O_2_, for R_C-O_ ≈ 2 Å. The two component transitions of this excited state as obtained with NTO are given with the corresponding coefficient, showing charge transfer from benzene to O_2_.

In order to describe the underlying molecular mechanism, several electronic excited states were computed in a frozen scan on the quasi-molecule [C_6_H_6_…O_2_]^+^. Figure 4b shows the potential energy curves of the 250 computed electronic states as a function of the benzene-oxygen distance. States below ~ 24 eV (referred to the collision O_2_^–^ + C_6_H_6_) lie under the second ionization threshold of benzene*.* Interestingly, we observe attractive curves, that correspond to a Coulomb-type attraction between the doubly positively charged benzene and the negatively charged oxygen in the entrance channel. These curves then collapse at a minimum close to ~ 2 Å. In order to further our knowledge about the collision dynamics, a more refined examination of the potential energy surfaces has been implemented. To this, we have performed relaxed scans in the ground state, where the charge is redistributed over the whole [C_6_H_6_…O_2_] ^−^ system, and in the excited state of the lowest energy showing a charge transfer character, i.e. where it still presents the nature of the entrance channel with the superoxide anion approaching the doubly ionized benzene [O_2_^−^ → C_6_H_6_^2+^]. Figure 4c shows the difference in electron density distribution between these two states. A major change is appreciated with an increase of electron population of the O_2_ antibonding π* molecular orbital, which is filled by an electron from the C_6_H_6_ occupied π molecular orbital. The Natural Transition Orbital (NTO) analysis shown in Fig. 5 confirms the charge transfer character of this state. The relaxed scans in both electronic states are shown in Fig. 4d, from which while in the excited state oxygen-carbon bonding appears as a barrier less process, a barrier of ~ 0.7 eV is shown in the ground state. Both calculations allow us to locate the corresponding minima in the potential energy surfaces, with strong covalent oxygen-carbon bonds stabilizing such structures, and the excited state showing charge transfer character. We can assume that non-radiative decay processes may be operative in such stabilization, since both states present similar structures and the corresponding potential energy curves are not far apart in energy, thus leading the system in the electronic ground state. It is precisely from this point where we continue our examination of the potential energy surface. Figure 4e shows the critical points, minima and transition states, in several reaction paths that lead to the production of stable diol structures (labeled as Min6 and Min8 in the figure). Energy barriers of ~ 2–3 eV in the forward direction, yielding diol structures, can be easily reached if we assume that the entrance channel is at higher energy. However, much higher energy barriers of ~ 7–8 eV prevent the diol structures to come back and to dissociate into O_2_/C_6_H_6_. Thus, these are kept trapped in these potential wells. Interestingly, these two diol structures appear only at ~ 3.7 eV above the initial point before the double ionization, i.e. C_6_H_6_ + O_2_^−^. Such great stability seems to indicate that the 110 u feature assigned to C_6_H_6_O_2_^+^ most likely corresponds to any of these two diol compounds. Nevertheless, these structures may carry enough internal energy to continue evolving through low-energy barrier paths, e.g. with the loss of OH; this is supported by the experiment where fragments 93 u assigned to C_6_H_5_O^+^ and/or 94 u to C_6_H_6_O^+^ are detected as combined features (see Fig. 3b) due to the lack of mass resolution. Complementary molecular dynamics studies on the C_6_H_6_O_2_^+^ compound (Min1), confirm the stability of this structure, thus verifying the validity of the proposed mechanism (see details in the Supplementary Information). The formation of the complex should be a fast process taking place in a fs time scale. Considering strong coulomb attraction between doubly ionized benzene and O_2_^−^, and assuming excitation energies of about 15-20 eV, a few tens of femtoseconds is enough to cleavage and form covalent bonds^27,28^.

It is worth mentioning in this point that benzene dication undergoes rearrangement to a more stable pyramidal isomer with a C_5_H_5_ base and CH at the apex^29^. It has been proposed that this structure is composed of a donor–acceptor bond from a C_5_H_5_^−^ to a CH^3+^ moiety^30^. The isomerization barrier between the dicationic canonical form of benzene and the pyramidal one is of 100 kJ·mol^−1^, but we consider that in our case this possibility can be ruled out, since the Coulomb attraction between C_6_H_6_^2+^ and O_2_^–^ leads to a very rapid formation of C_6_H_6_-O_2_^+^ complex.

A careful analysis of the experimental mass spectra can provide additional information on the observed peak composition. Note that the time (mass) resolution of the present experiment (Δ*m*/*m* ≅ 0.05) is limited by the gas cell configuration and the inherent characteristic of the hollow cathode discharge. Nonetheless, the decomposition of the observed peaks into single Gaussian functions, representing the different cationic fragment masses, may enlighten which ion species are contributing to the observed peaks. As mentioned above, Fig. 3b shows the difference between the anion and electron induced fragmentation spectra once normalized to the parent ion (C_6_H_6_^+^) intensity, and therefore features around 78 u do not appear in this plot. Similar conditions concur for the peaks around 50 u, i.e. C_4_H_*n*_^+^ (*n* = 0–5) ion fragments. However, there is a clear enhancement of the peaks around 60 u and 38 u, corresponding to C_5_H_*n*_^+^ (*n* = 1–6) and C_3_H_*n*_^+^ (*n* = 0–5), respectively, as well as new weaker structures appearing below 30 u. This suggests that anion collisions are favoring further parent ion fragmentation either by losing CH_*n*_ (*n* = 0–4) fragments or breaking it into two similar size fragments.

For ions corresponding to *m/z* > 78 u, in which this study is mainly focused, we found the aforementioned *m/z* = 110–120 u structure together with some smaller peaks around 94 and 99 u. The analysis of these structures using the decomposition into Gaussian functions reveals that *m/z* = 110–120 u is formed by the contribution of two features centered at 110 and 126 u, respectively. The 110 u is assigned to C_6_H_6_O_2_^+^ with its formation mechanism supported by theoretical calculations described above. The 126 u ion can be explained under the same formation rationale as the 110u feature but now being formed by the O_3_^−^ anion, also present in the projectile beam, as: double ionization of benzene is followed by interactions between O_3_^−^ and C_6_H_6_^2+^ leading to stable trihydroxybenzene (benzenetriol) structures C_6_H_6_O_3_^+^. O_3_^−^ is also formed in the hollow cathode discharge and, although its time of flight from the cathode to the scattering cell is longer than O_2_^−^, the uncertainty in position where they are formed along the discharge contributes to a contamination of the primary O_2_^−^ beam (see FIG. 2a). Finally, we must note that the small feature at 94 ± 2 u confirms that the C_6_H_6_O_2_^+^ product may have enough internal energy to further decompose by losing OH^•^ or O^•^ radicals thus yielding C_6_H_5_O^+^ or C_6_H_6_O^+^, respectively, as predicted by the quantum chemical calculations.

**Figure 6:**
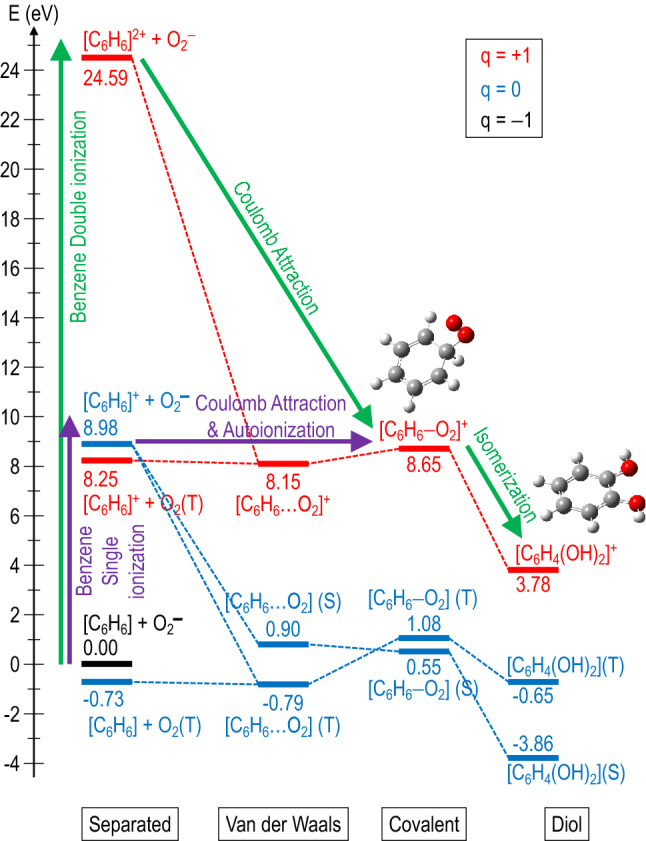
Different ways to produce neutral and positive charged benzene diols via O_2_^−^ collisions with C_6_H_6_; ―, single benzene ionization plus O_2_^−^ Coulomb attraction to produce neutral diol configurations; ―, same process but producing an autoionizing state which decay to the benzene diol cation. ―, double benzene ionization plus Coulomb attraction leading to benzene diol cation formation.

However, it is still surprising the high intensity of the *m/z* ~ 110–120 u structure in comparison with *m/z* = 78 u. Although double ionization of benzene has proven to be particularly likely^17^, single ionizing processes contributing to the 78 m*/z* peak should be even more pronounced. Nonetheless, this can be explained within our proposed model. Note that the Coulomb attraction between the formed cation and the anion projectile in the case of single ionization will give a neutral compound (C_6_H_6_O_2_) which will not be detected by our TOF spectrometer but will contribute to reduce the observed 78 m*/z* peak intensity. Thus, an alternative way for the formation of the 110–120 m*/z* structure can be considered if the neutral C_6_H_6_O_2_ compound is formed in an excited autoionizing state that finally decays to C_6_H_6_O_2_^+^, whose evolution would follow the potential energy surface shown in Fig. 4e. Note that the lifetime of this excited state will naturally contribute to form a broad structure as that shown here. In good agreement with the proposed model, the two pathways to form neutral and cation benzene diols via O_2_^−^ collisions with C_6_H_6_ are summarized in Fig. 6.

## Conclusions

In conclusion, we have experimentally shown the formation of large complex molecules (larger than the target molecule) in relatively high energy (200–900 eV) collisions of oxygen anions with neutral benzene molecules. Quantum chemical calculations have been performed within the framework of the density functional theory, to further our knowledge on the possible paths involved to generate these molecules. We have then proposed the formation of “quasi-molecular” compounds resulting from the electrostatic attraction between a doubly ionized target molecule and the negatively charged projectile. These electrostatically bonded complexes finally decay via non-radiative processes resulting in stable diol/triol structures in collisions with O_2_^−^/O_3_^−^. We have also shown that an alternative way to generate these complexes is to single ionize the benzene molecule, forming the “quasi-molecular” compound in an autoionizing state of its neutral configuration which finally decays to the corresponding diol cation. We can then confirm that the present experimental evidences are supported by the theoretical calculations. This model presents a new high energy anion-driven chemistry as an alternative way to form complex molecules.

## Supplementary Information

Supplementary Information.
