# Supplementary material for: Unexpected benzene oxidation in collisions with superoxide anions
Source: Sci Rep. 2021 Nov 30;11:23125. doi: 10.1038/s41598-021-02408-7 (PMC8633363; doi:10.1038/s41598-021-02408-7)
Supplement: Supplementary file 1 — Supplementary Information. [file 41598_2021_2408_MOESM1_ESM.pdf]

Supplementary Information for
Unexpected benzene oxidation in collisions with superoxide anions

Carlos Guerra[1], Sarvesh Kumar[2], Fernando Aguilar-Galindo[3], Sergio Díaz-Tendero[4,5,6,*], Ana I. Lozano[2], Mónica Mendes[2], Paulo Limão-Vieira[2], and Gustavo García[1,7,*]

[1]Instituto de Física Fundamental, Consejo Superior de Investigaciones Científicas, Serrano 113-bis, 28006 Madrid, Spain.
[2]Laboratório de Colisões Atómicas e Moleculares, CEFITEC, Departamento de Física, Faculdade de Ciências e Tecnologia, Universidade NOVA de Lisboa, 2829-516 Caparica, Portugal.
[3]Donostia International Physics Center (DIPC), Paseo Manuel de Lardizabal 4, Donostia-San Sebastián, E-20018, Spain
[4]Departamento de Química, Módulo 13, Universidad Autónoma de Madrid, 28049 Madrid, Spain
[5]Condensed Matter Physics Center (IFIMAC), Universidad Autónoma de Madrid, 28049 Madrid, Spain.
[6]Institute for Advanced Research in Chemical Science (IAdChem), Universidad Autónoma de Madrid, 28049 Madrid, Spain.
[7]Centre for Medical Radiation Physics, University of Wollongong, NSW, Australia.
*corresponding authors: sergio.diaztendero@uam.es and g.garcia@csic.es

Ab initio molecular dynamics

We have performed ab initio molecular dynamics using the Atom-centered Density Matrix Propagation (ADMP) method[1-3], an extended Lagrangian approach implemented in the Gaussian16 package[4]. We performed classical trajectory calculations with the electronic structure computed in the framework of the density functional theory (DFT), in particular with the M06-2X functional[5] and the 6-31++G(d,p) basis set[6-8]. This level of theory has shown to be adequate to accurately describe intermolecular interactions for compounds of the main groups, including non-covalent interactions, such as van der Waals forces and hydrogen bonds[9-10]. All simulations were performed using a time step of Δt=0.1fs and a fictitious electron mass of μ=0.1 amu, to ensure adiabaticity in the dynamics. To mimic the experimental conditions, we have introduced a certain amount of excitation energy. We considered three set of simulations using E_{exc}= 5, 15 and 25 eV. This range of excitation energy should cover the transferred energy in the collisions. We have considered a maximum propagation time for the trajectories of t_{max} = 200 fs. At this point, two atoms have been considered to be bonded if the distance between them is smaller than 3 Å; at larger distances we assume that the atoms belong to separate fragments. The charge in the corresponding fragment is obtained as the sum of the atomic charges in the fragment adopting the Mulliken population scheme[11].

This methodology has been employed starting with two possible conformers: a weakly bonded $[C_6H_6\cdots O_2]^+$ and a covalently bonded structure, Min1 as labelled in Figure 4e of the main article, both minima in the potential energy surface of the ground state. For each structure and each value of excitation energy, we considered 100 trajectory calculations and we computed statistics on the populated channels after the considered propagation time. Results are given in Tables S1 and S2, respectively. The results show the high instability of the weakly bonded structure, leading in all cases to O_2 emission and, at the highest excitation energy, also hydrogen abstraction or

emission. However, in the case of Min1, stable oxygenated species are observed after 200 fs, being the non-fragmented $C_6H_6O_2^+$ the most populated channel in the studied excitation energy range. Other channels leading to hydrocarbon structures are also populated for the highest excitation energy, although to a lower extent, such as 9% $C_5H_5^+$ or 9% $C_3H_3^+$. These findings agree with the experimental measurements (see mass spectra in Figure 3a of the main text), thus supporting the validity of the proposed mechanism: double ionization of benzene, followed by coulomb attraction between O_2^- and $C_6H_6^{2+}$, to finally decay in a non-radiative process towards the covalently bonded structure Min1. Figure S1 extends the information shown in Figure 4e of the main article, by including all the structures that we have found in the studied pathways of the potential energy surface.

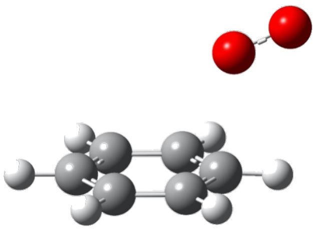

Table S1. $[C_6H_6\cdots O_2]^+$ weakly bonded. Percentage of the channels populated at t_{max}=200fs for each value of excitation energy.

Channel	5eV	15 eV	25 eV
$H_6C_6O_2^+$	1	0	1
O_2 / $C_6H_6^+$	99	100	62
H / $C_6H_5O_2^+$	0	0	1
HO_2 / $C_6H_5^+$	0	0	4
O/ OH / $C_6H_5^+$	0	0	1
HO_2 / H / $C_6H_4^+$	0	0	1
O_2 / H / $C_6H_5^+$	0	0	28
O_2 / H_2 / $C_6H_4^+$	0	0	2

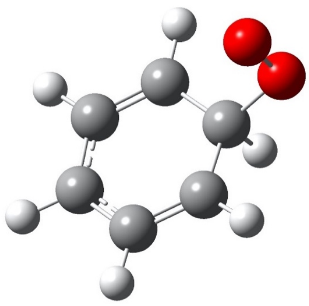

Table S2. Min1 $[C_6H_6O_2]^+$. Percentage of the channels populated at t_{max}=200fs for each value of excitation energy.

Channel	5eV	15 eV	25 eV
$H_6C_6O_2^+$	68	51	28
O_2 / $C_6H_6^+$	32	43	16
O / $H_6C_6O^+$	0	1	7
H / $C_6H_5O_2^+$	0	1	10
HO_2 / $C_6H_5^+$	0	1	4
HO / $C_6H_5O^+$	0	3	9
H_2 / $C_6H_4O_2^+$	0	0	1
H_2O / $C_6H_4O^+$	0	0	1
$C_2H_3O^+$ / C_4H_3O	0	0	1
O_2 / H_2 / $C_6H_4^+$	0	0	1
O_2 / H / $C_6H_5^+$	0	0	13
O_2 / C_3H_3 / $C_3H_3^+$	0	0	9
O / H / $C_6H_5O^+$	0	0	9
H / H / $C_6H_4O_2^+$	0	0	9
OH / CO / $C_5H_5^+$	0	0	9
OH / H_2 / $C_6H_3O^+$	0	0	2
C_2H_2 / CO / $C_3H_4O^+$	0	0	1
H / CO_2 / $C_5H_5^+$	0	0	2

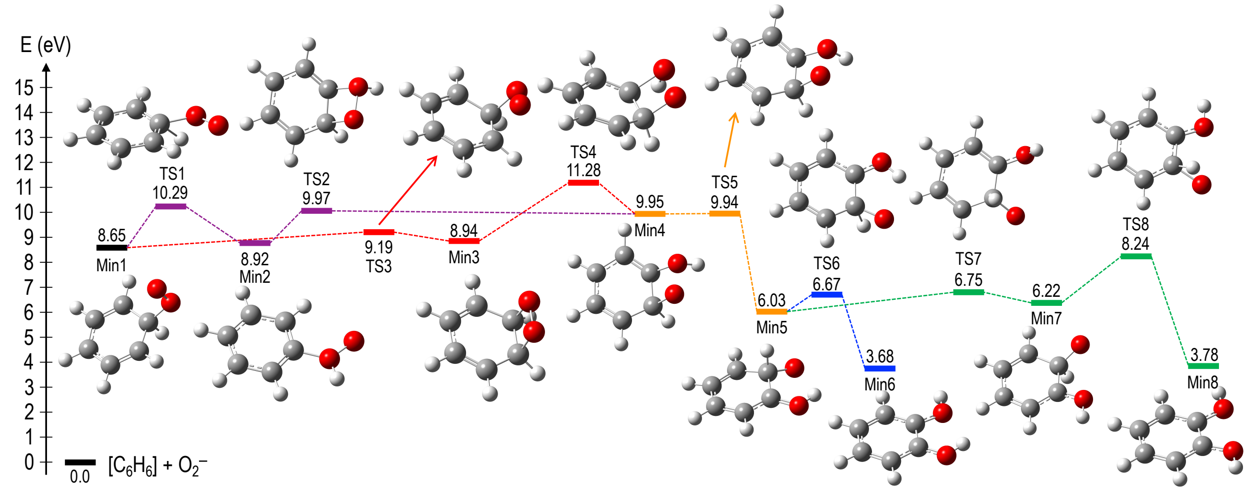

Figure S1. Critical points in the potential energy surface of the ground state of $C_6H_6O_2^+$. Min indicates a minimum, TS indicates a transition state. Relative energies are given in eV, corrected with the zero-point-energy (ZPE), and refereed to the ground state of C_6H_6 and O_2^- at infinite distance.

References

1. Iyengar, S. S.; Schlegel, H. B.; Millam, J. M.; A. Voth, G.; Scuseria, G. E.; Frisch, M. J. Ab Initio Molecular Dynamics: Propagating the Density Matrix with Gaussian Orbitals. II. Generalizations Based on Mass-Weighting, Idempotency, Energy Conservation and Choice of Initial Conditions. *J. Chem. Phys.* **2001**, *115* (22), 10291. https://doi.org/10.1063/1.1416876.
2. Schlegel, H. B.; Millam, J. M.; Iyengar, S. S.; Voth, G. A.; Daniels, A. D.; Scuseria, G. E.; Frisch, M. J. Ab Initio Molecular Dynamics: Propagating the Density Matrix with Gaussian Orbitals. *J. Chem. Phys.* **2001**, *114* (22), 9758–9763. https://doi.org/10.1063/1.1372182.
3. Schlegel, H. B.; Iyengar, S. S.; Li, X.; Millam, J. M.; Voth, G. A.; Scuseria, G. E.; Frisch, M. J. Ab Initio Molecular Dynamics: Propagating the Density Matrix with Gaussian Orbitals. III. Comparison with Born–Oppenheimer Dynamics. *J. Chem. Phys.* **2002**, *117* (19), 8694–8704. https://doi.org/10.1063/1.1514582.
4. Frisch, M. J.; Trucks, G. W.; Schlegel, H. B.; Scuseria, G. E.; Robb, M. A.; Cheeseman, J. R.; Scalmani, G.; Barone, V.; Petersson, G. A.; Nakatsuji, H.; Li, X.; Caricato, M.; Marenich, A. V.; Bloino, J.; Janesko, B. G.; Gomperts, R.; Mennucci, B.; Hratchian, H. P.; Ortiz, J. V.; Izmaylov, A. F.; Sonnenberg, J. L.; Williams-Young, D.; Ding, F.; Lipparini, F.; Egidi, F.; Goings, J.; Peng, B.; Petrone, A.; Henderson, T.; Ranasinghe, D.; Zakrzewski, V. G.; Gao, J.; Rega, N.; Zheng, G.; Liang, W.; Hada, M.; Ehara, M.; Toyota, K.; Fukuda, R.; Hasegawa, J.; Ishida, M.; Nakajima, T.; Honda, Y.; Kitao, O.; Nakai, H.; Vreven, T.; Throssell, K.; Montgomery, J. A., J.; Peralta, J. E.; Ogliaro, F.; Bearpark, M. J.; Heyd, J. J.; Brothers, E. N.; Kudin, K. N.; Staroverov, V. N.; Keith, T. A.; Kobayashi, R.; Normand, J.; Raghavachari, K.; Rendell, A. P.; Burant, J. C.; Iyengar, S. S.; Tomasi, J.; Cossi, M.; Millam, J. M.; Klene, M.; Adamo, C.; Cammi, R.; Ochterski, J. W.; Martin, R. L.; Morokuma, K.; Farkas, O.; Foresman, J. B.; Fox, D. J. *Gaussian 16, Revision C.01*; Wallingford CT, 2016. http://gaussian.com/
5. Zhao, Y.; Truhlar, D. G. The M06 Suite of Density Functionals for Main Group Thermochemistry, Thermochemical Kinetics, Noncovalent Interactions, Excited States, and Transition Elements: Two New Functionals and Systematic Testing of Four M06-Class Functionals and 12 Other Function. *Theor. Chem. Acc.* **2008**, *120* (1–3), 215–241. https://doi.org/10.1007/s00214-007-0310-x.
6. Hehre, W. J.; Ditchfield, R.; Pople, J. A. Self—Consistent Molecular Orbital Methods. XII. Further Extensions of Gaussian—Type Basis Sets for Use in Molecular Orbital Studies of Organic Molecules. *J. Chem. Phys.* **1972**, *56* (5), 2257–2261. https://doi.org/10.1063/1.1677527.
7. Hariharan, P. C.; Pople, J. A. The Influence of Polarization Functions on Molecular Orbital Hydrogenation Energies. *Theor. Chim. Acta* **1973**, *28* (3), 213–222. https://doi.org/10.1007/BF00533485.
8. Clark, T.; Chandrasekhar, J.; Spitznagel, G. W.; Schleyer, P. V. R. Efficient Diffuse Function-Augmented Basis Sets for Anion Calculations. III. The 3-21+G Basis Set for First-Row Elements, Li-F. *J. Comput.*

Chem. **1983**, *4* (3), 294–301. https://doi.org/10.1002/jcc.540040303.
9. Rousseau, P.; Piekarski, D.G.; Capron, M.; Comaracka, A.; Adoui, L.; Martín, F.; Alcamí, M.; Díaz-Tendero, S.; Huber, B.A. Polypeptide formation in clusters of beta-alanine amino acids by single ion impact. *Nat. Comm.* **2020**, *11*, 3818. https://doi.org/10.1038/s41467-020-17653-z.
10. Aguilar-Galindo, F.; Tuñón A.M.; Fraile, A.; Alemán, J.; Díaz-Tendero, S. Role of intramolecular hydrogen bonds and electron withdrawing groups in the acidity of aldimines and ketimines: a density functional theory study. *Theor. Chem. Acc.* **2019**, *138*, 59. https://doi.org/10.1007/s00214-019-2451-0.
11. Mulliken, R. S. Electronic Population Analysis on LCAO–MO Molecular Wave Functions. I. *J. Chem. Phys.* **1955**, *23* (10), 1833–1840. https://doi.org/10.1063/1.1740588.
